# Использование бесклеточного матрикса пуповины человека для замещения дефектов мягких тканей у пациента с синдромом диабетической стопы (описание клинического случая)

**DOI:** 10.14341/probl13615

**Published:** 2026-03-07

**Authors:** С. В. Чеботарёв, Д. В. Товпеко, Л. И. Калюжная, М. Е. Котова, А. А. Кондратенко, Д. А. Волов, В. В. Хоминец, В. Е. Чернов, Д. А. Земляной, В. В. Салухов

**Affiliations:** Военно-медицинская академия им. С.М. КироваРоссия; S.M. Kirov Military Medical AcademyRussian Federation; Военно-медицинская академия им. С.М. Кирова; Санкт-Петербургский государственный педиатрический медицинский университетРоссия; S.M. Kirov Military Medical Academy; Saint Petersburg State Pediatric Medical UniversityRussian Federation; Санкт-Петербургский государственный педиатрический медицинский университетРоссия; Saint Petersburg State Pediatric Medical UniversityRussian Federation

**Keywords:** синдром диабетической стопы, Вартонов студень пуповины человека, замещение дефектов мягких тканей, diabetic foot syndrome, Wharton’s jelly of the human umbilical cord, soft tissue defect replacement

## Abstract

Незаживающие язвы представляют серьезную проблему для общественного здравоохранения из-за высокой стоимости лечения, длительной нетрудоспособности пациентов и низкой эффективности терапии. Они являются ведущей причиной нетравматических ампутаций нижних конечностей.

Разработка и применение инновационных биоинженерных продуктов — значимый шаг для лечения незаживающих диабетических язв стопы. Одним из таких продуктов является тканеинженерный бесклеточный продукт из высокорегенеративного биоматериала пуповины человека. Он содержит факторы роста, цитокины и компоненты внеклеточного матрикса вартонова студня и ускоряет заживление ран.

ЦЕЛЬ данного клинического исследования — оценить безопасность и эффективность применения тканеинженерного бесклеточного продукта из Вартонова студня пуповины человека для лечения хронических незаживающих язв нижних конечностей у пациента с синдромом диабетической стопы.

**РЕЗУЛЬТАТЫ:**

РЕЗУЛЬТАТЫ: 60-летняя женщина с диагнозом «Сахарный диабет 2 типа» имела хронические язвы, которые более полутора лет не поддавались стандартному лечению. За ней наблюдали в течение 4 недель в августе 2024 года. В результате местного применения бесклеточного продукта из человеческой пуповины были отмечены признаки уменьшения размеров ран и их полное заживление через 4 недели после начала наблюдения.

**ЗАКЛЮЧЕНИЕ:**

ЗАКЛЮЧЕНИЕ: клиническое наблюдение продемонстрировало безопасность и эффективность бесклеточного продукта из человеческой пуповины для лечения хронических незаживающих язв диабетического происхождения.

## Abstract (en)

Non-healing ulcers pose a serious problem for public health due to the high cost of treatment, prolonged disability of patients, and low efficacy of therapy. They are the leading cause of non-traumatic lower limb amputations.

The development of innovative bioengineering products is a significant step in the treatment of non-healing diabetic foot ulcers. One such product is a tissue-engineered acellular product made from highly regenerative human umbilical cord biomaterial. It contains growth factors, cytokines, and components of the extracellular matrix of Wharton’s jelly, which accelerates wound healing.

THE AIM of this clinical study is to evaluate the safety and efficacy of a tissue-engineered acellular product from Wharton’s jelly of the umbilical cord for the treatment of chronic non-healing lower limb ulcers in patients with diabetic foot syndrome.

**RESULTS:**

RESULTS: a 60-year-old woman diagnosed with type 2 diabetes mellitus had chronic ulcers that had not responded to standard treatment for more than a year and a half. She was observed for 4 weeks in August 2024. As a result of the local application of an acellular product from human umbilical cord, signs of reduction in wound size and their complete healing were noted 4 weeks after the start of observation.

**CONCLUSION:**

CONCLUSION: clinical observation has demonstrated the safety and efficacy of the acellular product from human umbilical cord for the treatment of chronic non-healing ulcers of diabetic origin.

## Актуальность

Синдром диабетической стопы (СДС) представляет собой серьезное и часто инвалидизирующее осложнение сахарного диабета (СД), характеризующееся развитием язвенных поражений стоп с тенденцией к хроническому течению и затрудненному лечению [1]. Учитывая неуклонный рост распространенности диабета, по данным Всемирной организации здравоохранения, увеличивается и число пациентов с язвенными поражениями стоп [2]. При СД заживление дефектов мягких тканей замедляется из-за нейропатии, ишемии и повышенного риска инфицирования. В связи с этим возникает необходимость в поиске методов, способных минимизировать негативное влияние этих факторов. Традиционные методы лечения, включая медикаментозную терапию, хирургическую обработку ран и использование антисептиков, часто оказываются недостаточно эффективными для полного заживления [3].

В современной медицине существует потребность в новых терапевтических стратегиях, включая тканевую инженерию [4]. Использование тканеинженерных биоматериалов открывает возможности для лечения хронических язв, улучшения условий регенерации тканей и снижения риска инфекционных осложнений [5]. Одним из наиболее изученных подходов является применение аутологичных тромбоцитарных концентратов (PRP), богатых факторами роста, которые стимулируют ангиогенез, пролиферацию фибробластов и эпителизацию. Проведенные рандомизированные исследования показали, что применение PRP в виде локальных аппликаций способствует ускорению заживления хронических язв, снижая сроки эпителизации и риск инфицирования [6].

Другим направлением является использование стволовых клеток, прежде всего мезенхимальных стромальных клеток (МСК), полученных из костного мозга или жировой ткани. Они обладают способностью модулировать воспаление, стимулировать неоангиогенез и дифференцироваться в клетки соединительной ткани, способствуя репарации. Положительные результаты продемонстрированы как при локальном введении клеточных суспензий, так и при применении тканеинженерных матриксов, заселенных МСК [7].

Дополнительные перспективы открывает тканевая инженерия, включая создание кожных эквивалентов и биоматериалов с биологически активным покрытием. Использование децеллюляризованных матриц, биополимерных гидрогелей и носителей факторов роста обеспечивает структурную поддержку и направленную регенерацию [8].

Несмотря на обнадеживающие результаты, широкое применение этих технологий сдерживается высокой стоимостью, ограниченной доступностью сертифицированных препаратов и необходимостью стандартизации методов. Тем не менее регенеративные подходы к лечению мягкотканых дефектов при СДС постепенно входят в клиническую практику и могут существенно повысить эффективность лечения и снизить риск инвалидизации пациентов.

Особое внимание уделяется использованию внеэмбриональных человеческих тканей, таких как пуповина, которая демонстрирует высокий регенеративный потенциал [5]. Преимуществами таких материалов являются низкая иммуногенность и возможность использования без риска отторжения, что делает их безопасными и перспективными для пациентов с хроническими язвами СДС.

Богатый природный состав внеклеточного матрикса пуповины человека, содержащий большое количество сигнальных молекул и факторов роста, способствует более быстрому заживлению дефектов мягких тканей по сравнению с традиционными методами консервативного лечения [5]. Ускорение заживления происходит за счет стимуляции роста грануляционной ткани, улучшения микроциркуляции в области дефекта и уменьшения воспалительного процесса. Продукты из пуповины обладают высокой биосовместимостью и способствуют активной пролиферации клеток и ангиогенезу [5]. Их использование несет меньше рисков и последствий для пациентов по сравнению с применением синтетических или ксеногенных имплантатов, что особенно важно для пациентов с диабетом, у которых повышен риск инфекционных и воспалительных процессов.

Цель исследования — продемонстрировать опыт клинического применения бесклеточного матрикса из пуповины человека для лечения дефекта мягких тканей у пациентки с синдромом диабетической стопы.

Цель данного клинического исследования — оценить безопасность и эффективность применения тканеинженерного бесклеточного продукта из Вартонова студня пуповины человека для лечения хронических незаживающих язв нижних конечностей у пациента с синдромом диабетической стопы.

Продукт из пуповины для лечения дефектов кожи и мягких тканей был разработан и запатентован в научно-исследовательском центре Военно-медицинской академии имени С.М. Кирова (Санкт-Петербург, Российская Федерация) [9][10].

## Описание случая

У пациентки П. 1964 года рождения установлен диагноз: «Сахарный диабет 2 типа» в 2009 году. Назначено лечение: глибенкламид по 5 мг два раза в день, метформин по 1000 мг два раза в день. Самоконтроль уровня гликемии натощак — 7–10 ммоль/л, постпрандиальную гликемию пациентка не контролировала. В 2019 г. выполнена ампутация второго пальца правой стопы из-за синдрома диабетической стопы, осложненного гангреной. В 2023 г. пациентка заметила появление язвенных дефектов на правой стопе (ссадины от обуви), лечилась самостоятельно: перевязки с мазями «Левомеколь» и «Аргосульфан» — без положительного эффекта.

Во время первой госпитализации в клинику при осмотре правой стопы (июль 2023 г.): отсутствует второй палец, ампутированный в 2019 г. из-за осложнений СДС. На подошвенной поверхности стопы отмечается гиперкератоз. Визуализируются дефекты мягких тканей в области подошвенной поверхности первого пальца и первого плюснефалангового сустава правой стопы. Дефект округлой формы, диаметром около 1,5–2 см, с неровными краями и признаками гиперкератоза вокруг. Дно дефекта покрыто грануляциями, умеренно влажное, с серозным отделяемым, без признаков нагноения (рис. 1). Покровы стопы умеренно гиперемированы, кожа сухая, с признаками снижения тургора. Пальпация первого плюснефалангового сустава безболезненная, в области головок плюсневых костей имеются плотные мозоли.

**Figure:**
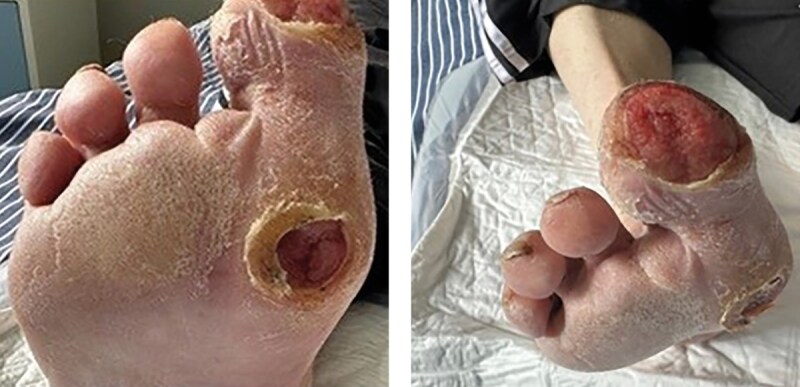
Рисунок 1. Внешний вид правой стопы при поступлении в клинику (июль 2023 г.).

На снимках рентгенографии правой стопы в двух проекциях выявлены признаки дегенеративно-дистрофических изменений и остеоартроза (рис. 2). Уменьшена ширина суставных щелей первого плюснефалангового сустава и межфалангового сустава первого пальца, наблюдается субхондральный склероз и заострение суставных поверхностей. Эти изменения могут быть обусловлены нарушениями кровообращения и хроническим воспалением, характерными для диабетической нейропатии и ангиопатии, и они усугубляют состояние суставов и костной ткани при СДС.

**Figure:**
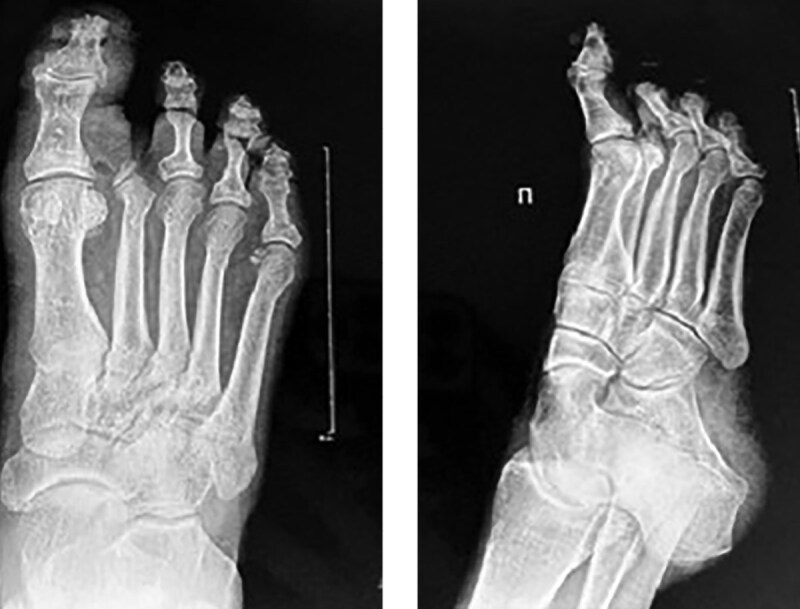
Рисунок 2. Рентгенограмма правой стопы в двух проекциях. Уменьшена ширина суставных щелей первого плюснефалангового сустава и межфалангового сустава первого пальца, наблюдается субхондральный склероз и заострение суставных поверхностей.

Магнитно-резонансная томография правой стопы выявила трабекулярный отек костного мозга дистальной фаланги первого пальца (рис. 3). Этот отек может свидетельствовать о воспалительном процессе или процессе асептического некроза, что типично для пациентов с СДС, у которых часто наблюдаются нарушения микроциркуляции.

**Figure:**
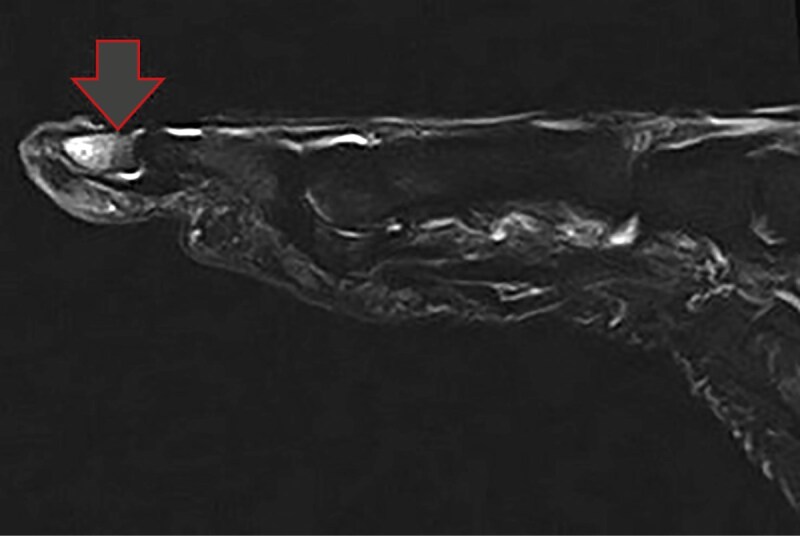
Рисунок 3. Магнитно-резонансная томография правой стопы. Трабекулярный отек костного мозга дистальной фаланги первого пальца.

Результаты лабораторных анализов: уровень глюкозы в крови натощак — 12,4 ммоль/л (целевой показатель — менее 7,5 ммоль/л); уровень глюкозы в крови после приема пищи — 8,2–12 ммоль/л (целевой показатель — менее 10 ммоль/л); гликированный гемоглобин (HbA1c) — 8,4% (целевой показатель — менее 7,5%); клинический анализ крови — в норме; С-реактивный белок — в норме; дислипидемия: общий холестерин — 7,13 ммоль/л (целевой показатель — менее 4 ммоль/л), триглицериды — 1,79 ммоль/л (целевой показатель — менее 1,7 ммоль/л), липопротеины низкой плотности — 4,42 ммоль/л (целевой показатель — менее 1,4 ммоль/л); скорость клубочковой фильтрации — 55 мл/мин/1,73 м²; отношение альбумина к креатинину в моче — 245,4 мг/г креатинина (<30 мг/г креатинина); микроальбуминурия — 245,4 (0–30) мг/мл.

Обследование показало необходимость коррекции гипогликемической терапии, улучшения контроля уровня липидов и возможного изменения тактики лечения для предотвращения прогрессирования заболевания.

Дуплексное сканирование артерий нижних конечностей: стенки артерий не утолщены, уплотнены, просвет свободен, кровоток магистральный на всех уровнях. Заключение: ультразвуковых признаков стеноза и окклюзий основных артерий нижних конечностей нет. Аnkle-brachial index — 1,12 (норма — 0,9–1,3).

Микробиологическое исследование содержимого ран правой стопы выявило Escherichia coli в концентрации 10⁷ КОЕ/мл и Enterococcus faecalis в концентрации 10⁴ КОЕ/мл. Это потребовало проведения антибактериальной терапии с учетом чувствительности выявленных микроорганизмов для эффективного лечения инфекционного осложнения при СДС.

Диагноз: cахарный диабет 2 типа. Синдром диабетической стопы, нейропатическая форма. Инфицированная язва первого пальца правой стопы и первого плюснефалангового сустава. Хроническая болезнь почек, характеризующаяся умеренно сниженной скоростью клубочковой фильтрации и высоким уровнем альбуминурии. Сопутствующие заболевания: гипертоническая болезнь II стадии, контролируемая артериальная гипертензия, группа риска — очень высокий. Дислипидемия, характеризующаяся высокой атерогенностью (высокие уровни холестерина, триглицеридов, липопротеинов низкой плотности и очень низкой плотности).

Во время обработки раневой области правой стопы была удалена некротическая ткань (рис. 4).

**Figure:**
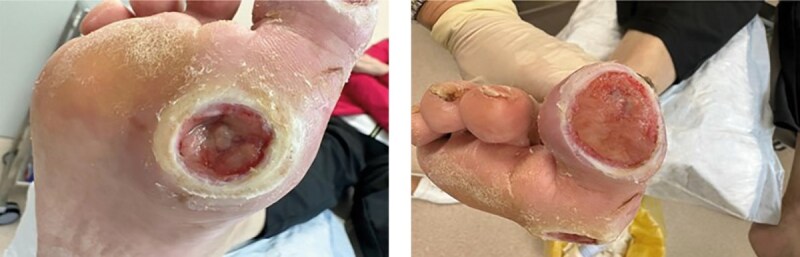
Рисунок 4. Вид правой стопы после тщательной и щадящей хирургической обработки раны.

Была скорректирована терапия: специализированная диета, разработанная для пациентов с диабетом 2 типа, направленная на поддержание стабильного уровня сахара в крови и предотвращение скачков уровня глюкозы; гипогликемическая терапия: метформин — 1000 мг два раза в день, эмпаглифлозин — 25 мг утром; розувастатин — 20 мг в день; антибактериальная терапия: моксифлоксацин внутривенно — 400 мг в день в течение 10 дней, затем амикацин внутримышечно — 500 мг в течение 21 дня; перевязки с использованием липид-коллоидных раневых повязок каждый день; на ноги была наложена индивидуальная полимерная разгрузочная повязка (тотал-контактный гипс).

Пациентка не всегда соблюдала рекомендации, в частности, иногда передвигалась без использования костылей, допуская нагрузку на правую стопу. Стационарное лечение с периодической хирургической обработкой ран сопровождалось небольшим уменьшением размеров ран и увеличением гиперкератоза и сухости кожи вокруг раны (рис. 5).

**Figure:**
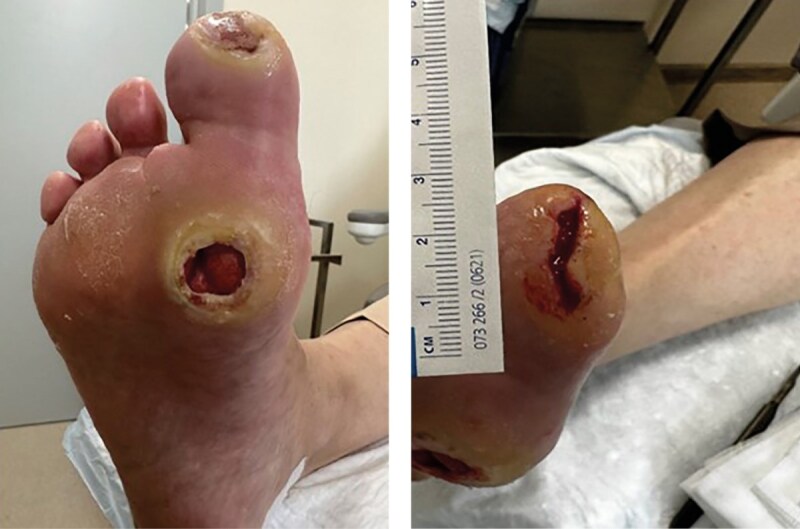
Рисунок 5. Динамика заживления ран (август 2023 г.).

Наблюдалось частичное заживление ран на пальцах, которое было достигнуто за счет снижения физической активности при ходьбе (рис. 6).

**Figure:**
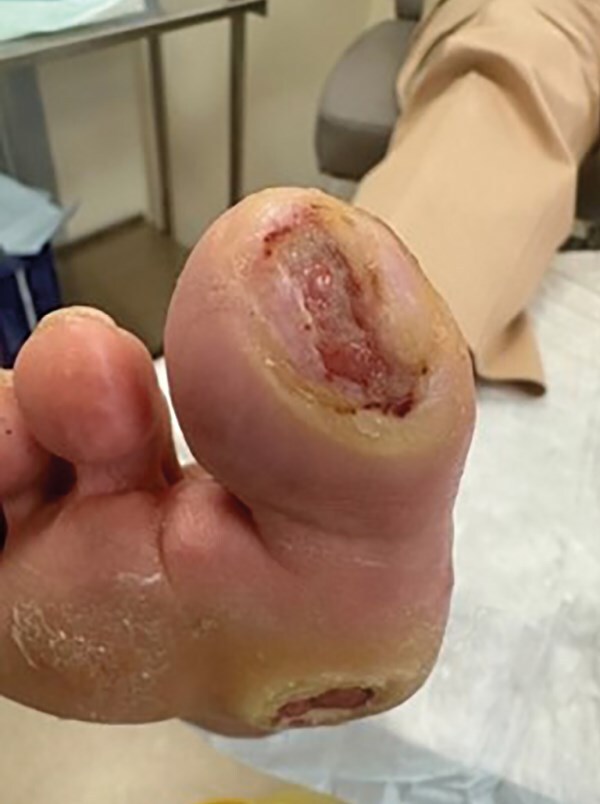
Рисунок 6. Динамика заживления ран (ноябрь 2023 г.).

При восстановлении обычного физиологического режима ходьбы были отмечены усиление гиперемии дна раны и шероховатость кожи вокруг раны, что вызывало напряжение и дискомфорт у пациентки (рис. 7 А, Б).

**Figure:**
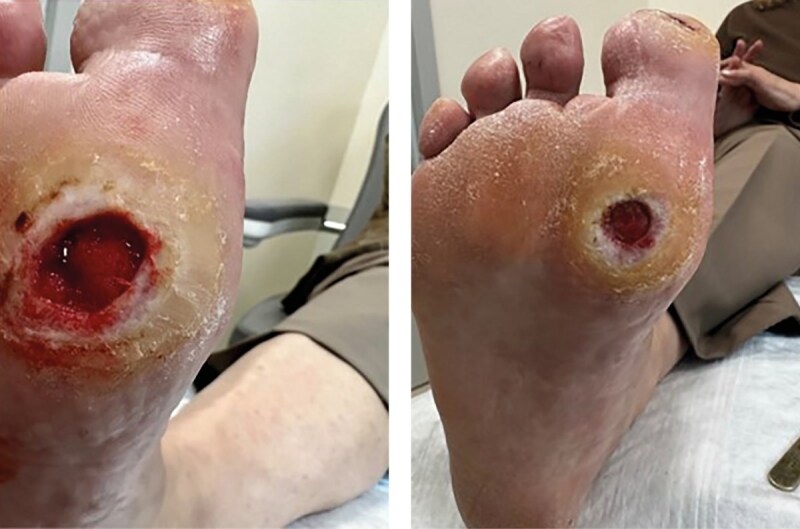
Рисунок 7. Динамика заживления ран (январь 2024 г.).

При осмотре пациентки в мае 2024 г. признаки заживления дефектов мягких тканей в области подошвенной поверхности первого пальца и первого плюснефалангового сустава правой стопы не вывялены. Края раны оставались неровными и плотными, с признаками гиперкератоза по периферии. Объем грануляционной ткани минимален, наблюдались застойные изменения, указывающие на низкую регенеративную активность. Несмотря на проводимую терапию, уменьшения размеров дефекта и эпителизации не происходило. Дно раны оставалось влажным, с серозным отделяемым, количество которого со временем не уменьшалось, что свидетельствовало о продолжающемся воспалительном процессе (рис. 8).

**Figure:**
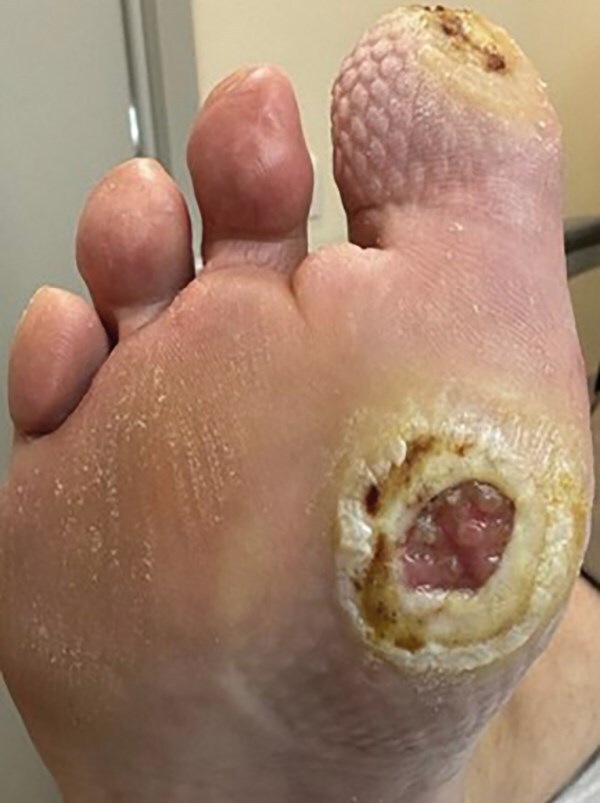
Рисунок 8. Динамика раневого процесса (май 2024 г.). Выраженный гиперкератоз вокруг раневой области первого плюснефалангового сустава.

В июне 2024 г. для стимуляции ангиогенеза и эпителизации был использован лиофилизированный бесклеточный продукт из ткани пуповины человека. Применение продукта было одобрено согласно «Протоколу этического комитета» SF_LEK_MT_0702 от 06.06.2022 (рис. 9).

**Figure:**
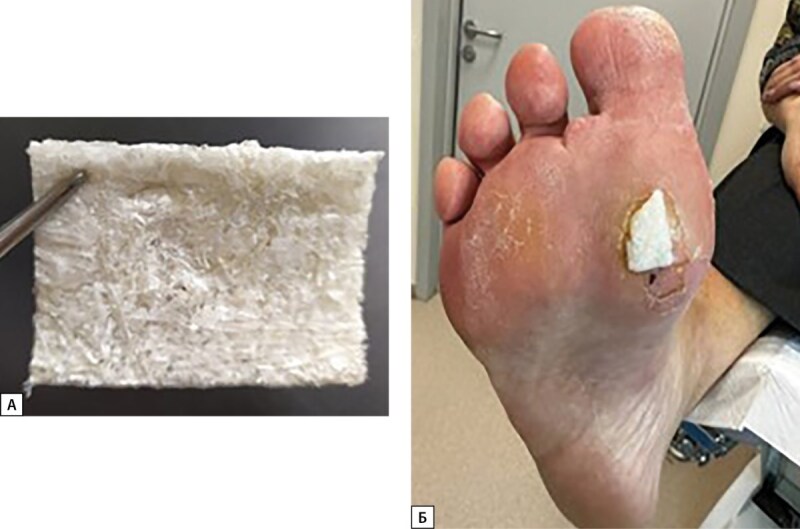
Рисунок 9. А — бесклеточная матрица из пуповины человека, Б — перевязка раны с использованием бесклеточной матрицы из пуповины человека.

Продукт из пуповины применялся при каждой перевязке (один раз в 3–4 дня) в течение 3 недель. Использовались асептические повязки без применения дополнительных фармакологических средств для закрытия ран, вокруг дефекта мягких тканей накладывалась фетровая разгрузочная повязка (рисунок 10 А, Б).

**Figure:**
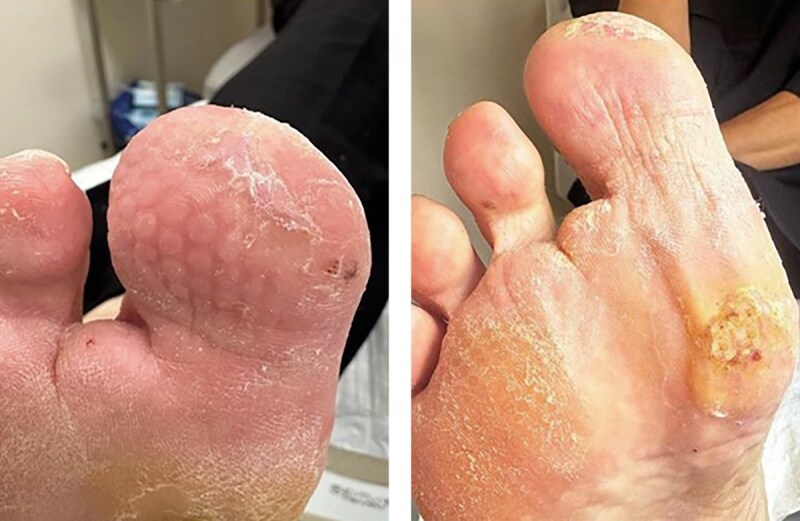
Рисунок 10. Динамика заживления ран после использования бесклеточной матрицы из пуповины человека (конец августа 2024 г.): диабетическая рана большого пальца полностью покрыта эпителием, раневая область первого плюснефалангового сустава эпителизирована, гиперкератоз менее выражен.

## Обсуждение

Представленный случай лечения пациента с успешным заживлением язв при синдроме диабетической стопы примечателен с точки зрения применения нового подхода к местной терапии ран — использования бесклеточной матрицы из пуповины человека.

С момента начала лечения продуктом из пуповины началось активное формирование грануляционной ткани. Дно раны постепенно заполнялось грануляциями насыщенного розового цвета, что указывало на высокую регенеративную активность тканей. Происходила постепенная эпителизация и уменьшение размеров раны. Края раневой поверхности становились более гладкими и мягкими, что свидетельствовало о снижении гиперкератоза.

Использование бесклеточного продукта из пуповины человека способствовало активации ангиогенеза, о чем свидетельствовало появление множества мелких новообразованных кровеносных сосудов в грануляционной ткани, что улучшило трофику пораженной области и способствовало ускорению регенеративных процессов.

Воспалительные проявления, гиперемия вокруг раны и боль при пальпации значительно уменьшились. Предположительно, механизм этих изменений заключается во влиянии факторов роста и компонентов тканеинженерного продукта из пуповины на стимуляцию миграции стволовых клеток в очаг воспаления. В экспериментах на грызунах и свиньях мы показали колонизацию бесклеточной тканеинженерной конструкции клетками реципиента. Высокая гигроскопичность продукта способствовала его фиксации в ране. В наших экспериментальных исследованиях продукт поглощал жидкость в 16 раз больше исходной массы образца [5].

Асептические повязки помогали поддерживать оптимальные условия влажности и стерильности, что также положительно влияло на процесс заживления. Постепенно размеры ран уменьшались, раны покрывались новообразованным эпителием, что свидетельствовало об эффективной репарации поврежденных тканей.

Таким образом, использование лиофилизированного бесклеточного продукта из пуповины человека продемонстрировало выраженный положительный эффект в заживлении дефектов мягких тканей, улучшении процессов регенерации, трофики и снижении воспалительных проявлений.

Пациент продолжает адекватную гипогликемическую, липидоснижающую, ангиопротекторную и антигипертензивную терапию.

В дальнейшем планируется проведение проспективного клинического исследования для оценки эффективности и воспроизводимости данного метода на расширенной выборке пациентов.

## Заключение

Применение лиофилизированного продукта из пуповины человека демонстрирует высокую эффективность в лечении диабетических ран, ускоряя эпителизацию, снижая гиперкератоз окружающих тканей и не вызывая местных аллергических, инфекционных или воспалительных реакций. Зона заживления функционально сопоставима с неповрежденной тканью, что, вероятно, связано с наличием в продукте факторов роста, цитокинов, молекул адгезии и компонентов фетального фенотипа, подтвержденным в наших предыдущих экспериментальных работах. Учитывая высокий риск рецидивов у пациентов с диабетической стопой, критически важными остаются регулярное использование ортопедической обуви и индивидуальных стелек, а также динамическое наблюдение в специализированном кабинете диабетической стопы для предотвращения осложнений и поддержания долгосрочного результата лечения.

## Дополнительная информация

Источники финансирования. Работа выполнена по инициативе авторов без привлечения финансирования.

Конфликт интересов. Авторы декларируют отсутствие явных и потенциальных конфликтов интересов, связанных с содержанием настоящей статьи.

Участие авторов. Все авторы одобрили финальную версию статьи перед публикацией, выразили согласие нести ответственность за все аспекты работы, подразумевающую надлежащее изучение и решение вопросов, связанных с точностью или добросовестностью любой части работы.

Согласие пациента. Авторы настоящей статьи получили письменное разрешение от упоминаемых в статье пациентов на публикацию их медицинских данных в журнале «Проблемы эндокринологии».
